# Knockdown of *myorg* leads to brain calcification in zebrafish

**DOI:** 10.1186/s13041-022-00953-4

**Published:** 2022-07-23

**Authors:** Miao Zhao, Xiao-Hong Lin, Yi-Heng Zeng, Hui-Zhen Su, Chong Wang, Kang Yang, Yi-Kun Chen, Bi-Wei Lin, Xiang-Ping Yao, Wan-Jin Chen

**Affiliations:** Department of Neurology, Institute of Neurology of First Affiliated Hospital, Institute of Neuroscience, and Fujian Key Laboratory of Molecular Neurology, Fujian Medical University, Fuzhou, 350005 China

**Keywords:** Primary familial brain calcification, *Myorg*, Zebrafish, Antisense oligo, Knockdown

## Abstract

Primary familial brain calcification (PFBC) is a neurogenetic disorder characterized by bilateral calcified deposits in the brain. We previously identified that *MYORG* as the first pathogenic gene for autosomal recessive PFBC, and established a *Myorg*-KO mouse model. However, *Myorg*-KO mice developed brain calcifications until nine months of age, which limits their utility as a facile PFBC model system. Hence, whether there is another typical animal model for mimicking PFBC phenotypes in an early stage still remained unknown. In this study, we profiled the mRNA expression pattern of *myorg* in zebrafish, and used a morpholino-mediated blocking strategy to knockdown *myorg* mRNA at splicing and translation initiation levels. We observed multiple calcifications throughout the brain by calcein staining at 2–4 days post-fertilization in *myorg-*deficient zebrafish, and rescued the calcification phenotype by replenishing *myorg* cDNA. Overall, we built a novel model for PFBC via knockdown of *myorg* by antisense oligonucleotides in zebrafish, which could shorten the observation period and replenish the *Myorg*-KO mouse model phenotype in mechanistic and therapeutic studies.

**Supplementary Information:**

The online version contains supplementary material available at 10.1186/s13041-022-00953-4.

## Introduction

Brain calcification is often observed in the elderly. Its prevalence is higher in individuals with Parkinson’s disease, Alzheimer’s disease, and Down’s syndrome [1, 2]. The prevalence of brain calcification is estimated to be 6.6 per 1000 or higher in China [3]. Primary familial brain calcification (PFBC), also known as idiopathic basal ganglia calcification (IBGC) or Fahr’s disease, is a genetic neurodegenerative disorder that features bilateral symmetric brain calcification most prominently in the basal ganglia, thalamus, cerebellum, and subcortical white matter [4]. Among those tissues, the basal ganglia are the most likely to be affected, especially the globus pallidus. The clinical manifestations of PFBC vary and include motor disorders, psychiatric symptoms, cognitive impairment, seizures, migraine, dizziness, and can even be asymptomatic [5]. The brain calcification of PFBC autopsy samples detected by electron microscopy indicated that deposits were composed of a mixture of glycoproteins, mucopolysaccharides, calcium salts, and iron [6].

In recent years, six pathogenic genes have been identified for PFBC: *SLC20A2*, *PDGFRB*, *PDGFB*, *XPR1*, *MYORG*, and *JAM2* [7–12]. Of the six causative genes, *SLC20A2* and *XPR1* are both phosphate transporters that play prominent roles in maintaining Ca-P homeostasis [13, 14]. *PDGFRB* and *PDGFB* encode a receptor and ligand in the mural cells (pericytes and smooth muscle cells) and endothelial cells which are associated with blood-brain barrier (BBB) integrity [15]. MYORG, a putative glycosidase, was specifically located in astrocytes. [11]. Moreover, *JAM2* encodes a protein participating in a tight junction in the endothelial cells [16].

We first reported that biallelic *MYORG* mutations are the cause of autosomal recessive PFBC [11]. Clinically, PFBC patients with *MYORG* mutations mostly suffer from motor disorders (such as dysarthria, dysphagia, dystonia, parkinsonism, and ataxia), cognitive impairment, psychiatric symptoms, seizures, and dizziness. Their brain CT images consistently showed bilateral calcifications in the basal ganglia, dentate nucleus of cerebellum, subcortical white matter, and brainstem, etc. *MYORG* was originally identified from a proteomic analysis as a nuclear envelope transmembrane protein with glycosidase homology [17]. *MYORG* is also necessary for myogenic differentiation [18, 19]. We next detected that *Myorg* mRNA is largely localized to astrocytes, particularly in the Bergmann glia [11]. Brain calcification was observed in the thalamus of the *Myorg* knockout (KO) mouse model from approximately nine months of age, mimicking the phenotype in the PFBC patients. However, as a neurodegeneration progress, the *Myorg*-KO mouse models are difficult to study since brain calcification requires long observation periods, hindering the mechanistic studies of PFBC caused by loss of function of *MYORG*.

In this study, we applied a knockdown strategy to *myorg* by antisense oligonucleotides (ASO) in zebrafish. This model led to the development of calcified deposits in the brain, which could be mitigated by *myorg* cDNA supplement. In this case, the ASO-mediated knockdown strategy of *myorg* in zebrafish is another model for phenotypically and mechanistically studying PFBC, and possibly other neurodegenerative diseases.

## Materials and methods

### Zebrafish care and maintenance

Adult wild-type AB strain zebrafish were maintained at 28.5 ℃ on a 14/10 h light/dark cycle. Five to six pairs of zebrafish were paired for natural mating every generation. On average, 200–300 embryos were generated. Embryos were maintained at 28.5℃ in fish water (0.2% Instant Ocean Salt in deionized water). The embryos were washed and staged according to standard procedures [20]. The zebrafish facility at Shanghai Research Center for Model Organisms is accredited by the Association for Assessment and Accreditation of Laboratory Animal Care (AAALAC) International.

### Whole-mount in situ hybridization

The primers for the *myorg* and control probes produced from cDNA of zebrafish brain are as follows: *myorg-*pb*-*F: 5′-ATGGGAGTATGATGATGAGGTT-3′, *myorg-*pb*-*R: 5′-TAATACGACTCACTATAGGTTAAATGCAAGGCACGGA-3′, control-pb-F: 5′-TAATACGACTCACTATAGGATGGGAGTATGATGATGAGGTT-3′, control-pb-R: TTAAATGCAAGGCACGGA. This PCR product was transcribed for the digoxigenin (DIG)-labeled *myorg* RNA probe. The brains of adult zebrafish were isolated and fixed with 4% PFA, and used for the whole-mount in situ hybridization as previously reported [21].

### qPCR analysis

Total RNA was isolated from adult zebrafish brains using the Trizol method. The RNA was reverse-transcribed to cDNA using a NovoScript^®^ 1st Strand cDNA Synthesis SuperMix kit. qPCR was performed with a ChamQ SYBR qPCR Master Mix using Real-Time PCR System (Archimed, TMX4) with the following primers: *myorg*-RT-F: 5′-TGAAATGGTGAAGCCTAAAGAC-3′, *myorg*-RT-R: 5′-CCTGTTATGAAAGGTTTGGGTG-3; *ef1α*-RT-F: 5′-CTTCTCAGGCTGACTGTGC-3′, *ef1α*-RT-R: 5′-CCGCTAGCATTACCCTCC-3′. The data were analyzed with the baseline of Ct values, and each sample was measured with three replicates. And the relative *myorg* mRNA level was normalized to the *ef1α*.

### MO microinjections and rescue assay

Gene Tools, LLC (http://www.gene-tools.com/) designed the morpholino (MO). Antisense MO (Gene Tools) were microinjected into fertilized one-cell stage embryos according to standard procedures [22]. The morpholino experiments were performed according to previously reported guidelines [23]. The sequences of the *myorg* splice-blocking sequence were 5′-TAAGCACCATCCATACTGACCTGAA-3′ (*myorg*-E2I2-MO), the *myorg* translation-blocking morpholinos were 5′-AGGCACTACCTGGTACATTCTGAAC-3′ (*myorg*-ATG-MO), and the standard control morpholinos were 5′-CCTCTTACCTCAGTTACAATTTATA-3′ (Control-MO). The amount of the MO used for injection was 4 ng per embryo. Total RNA was extracted from 30 to 50 embryos per group using TriPure Isolation Reagent (Roche) according to the manufacturer’s instructions. RNA was reverse-transcribed to cDNA using a PrimeScript RT Reagent Kit with gDNA Eraser (Takara). Primers spanning *myorg* Exon 1 (forward primer: 5′-ACACGAAACCAACAGTCCTC-3′) and Exon 3 (reverse primer: 5′-TCCTTTGGCTTCACTGTCATAA-3′) of *myorg* were used for transcript analysis to confirm the efficacy of the *myorg*-E2I2-MO. The primer *ef1α* sequences used as the internal control were 5′-GGAAATTCGAGACCAGCAAATAC-3′ (forward) and 5′-GATACCAGCCTCAAACTCACC-3′ (reverse).

For the rescue assay, 4 ng *myorg*-E2I2-MO was co-injected with 50 pg pcDNA3.1 containing zebrafish *myorg* cDNA per embryo, respectively. The coding region of the wild-type zebrafish *myorg* (ENSDART00000157837.2) was synthesized by Sangon Biotech and subcloned into the pcDNA3.1 vector (Invitrogen).

### Calcein staining

Injected embryos were grown in 0.003% 1-phenyl-2-thiourea (PTU, Sigma, St. Louis, MO, USA) to block pigmentation and facilitate visualization until four days post-fertilization (dpf). After the treatment, zebrafish embryos (2–4 dpf) were washed three times with fish water and immersed in 0.2% calcein solution (C0875, Sigma-Aldrich) for 10 min. Next, zebrafish were thoroughly rinsed three times in fish water (5 min/wash) and anesthetized with 0.016% MS-222 (tricaine methanesulfonate, Sigma-Aldrich, St. Louis, MO, USA). The zebrafish were then oriented on their dorsal and lateral side and mounted with 3% methylcellulose (Sigma-Aldrich, St. Louis, MO, USA) in a depression slide for observation by fluorescence microscopy. The fluorescent chromophore, Calcein (C_30_H_26_N_2_O_13_), specifically binds to calcium, fluorescently staining the calcified structures in living zebrafish larvae and juveniles. This allowed us to analyze brain calcifications in live zebrafish with high sensitivity [24]. Zebrafish brain calcifications were also visible as bright green spots. The number of brain calcifications was quantitatively analyzed at different time points.

### Image acquisition

Embryos and larvae were analyzed using a Nikon SMZ 1500 Fluorescence microscope and subsequently photographed with digital cameras. A subset of images was adjusted for levels, brightness, contrast, hue, and saturation with Adobe Photoshop 7.0 software (Adobe, San Jose, CA, USA) to optimally visualize the expression patterns. Positive signals were defined by manually counting the number of green punctae using Image J. Ten zebrafish for each treatment were quantified, and we calculated the averaged total signal per animal.

### Statistical analysis

All data were presented as mean ± SEM. Statistical analysis and graphical representation of the data were performed using GraphPad Prism 7.0 (GraphPad Software, San Diego, CA, USA). Statistical significance was performed using a Student’s *t*-test, where appropriate. Statistical significance is indicated by *P < 0.05, **P < 0.01, ***P < 0.001, and ****P < 0.0001.

## Results

### *myorg* expression analysis in zebrafish

**Fig. 1:**
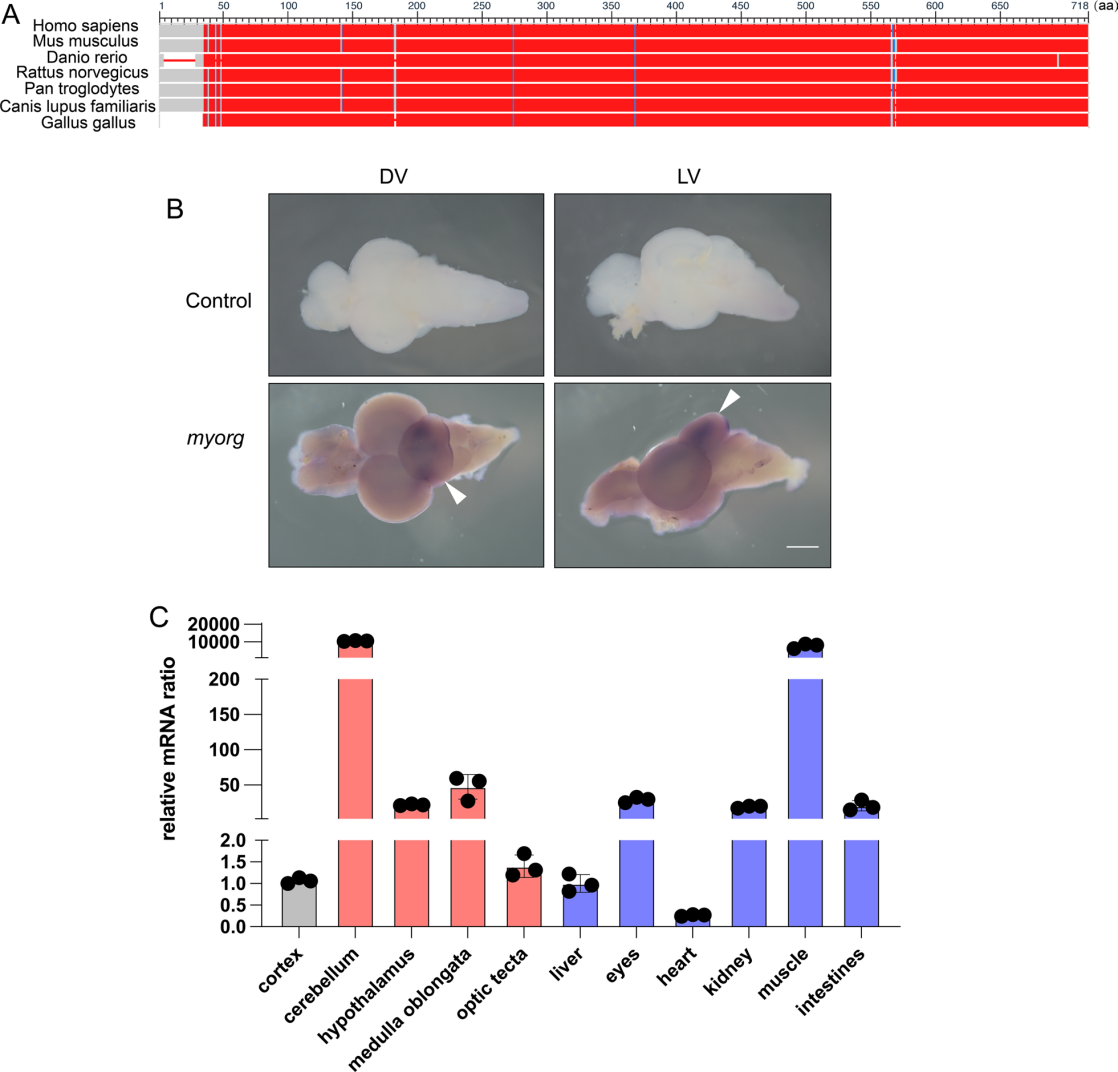
Expression pattern of *myorg* in zebrafish. **A** Sequence analysis of homology of amino acids of MYORG among different species (https://www.ncbi.nlm.nih.gov/gene/57462/ortholog/?scope=7776&term=MYORG). The color code legends could be found at the NCBI database (https://www.ncbi.nlm.nih.gov/tools/msaviewer/tutorial1/#conservation). **B** Whole-mount in situ hybridization analysis of *myorg* in the zebrafish brain. White arrows indicate the regions of positive signals. **C** The quantitative real-time PCR analysis of *myorg* in different tissues in zebrafish. Means ± SEM, n ≥ 3, Student’s *t*-test. Scale bar, 50 μm. *DV* dorsal view, *LV* lateral view

The myorg protein sequence shows about 67%~68% identity between the zebrafish and human or mice using BLAST, which could be regarded as average to high conservation between those species. (Fig. 1A). To analyze the *myorg* expression pattern in zebrafish, we first performed the whole-mount in situ hybridization and quantitative mRNA analysis of the zebrafish brain. *myorg* mRNA was highly expressed in the cerebellum in the central nervous system (Fig. 1B, C) in zebrafish, while *myorg* was most highly expressed in the muscle in the peripheral tissues (Fig. 1C). Also, *myorg* mRNA exhibited relative abundant expression in the hypothalamus, medulla oblongata, eyes, kidney, and intestine, but had low expression levels in the heart (Fig. 1C; Additional file 1: Table S1). The *myorg* expression pattern in zebrafish was similar to that of mice, especially in the brain [11].

As the Expression Atlas database (https://www.ebi.ac.uk/gxa/home) displayed, *myorg* is highly expressed in the development periods from blastula to larval day 5 except for few stages (like segmentation 14–17 somites and pharyngula prim-25) (Additional file 1: Fig. S1). A single-cell transcriptome atlas for zebrafish development suggested that the distribution of *myorg* RNA ranged from different cell types, including neuroblasts, neuron, cranial neural crest, and glial cells [25]. Collectively, *myorg* expressed broadly in the brain of zebrafish, including the critical regions, stages, and cell types.

### Calcification deposits in *myorg* knockdown zebrafish

**Fig. 2:**
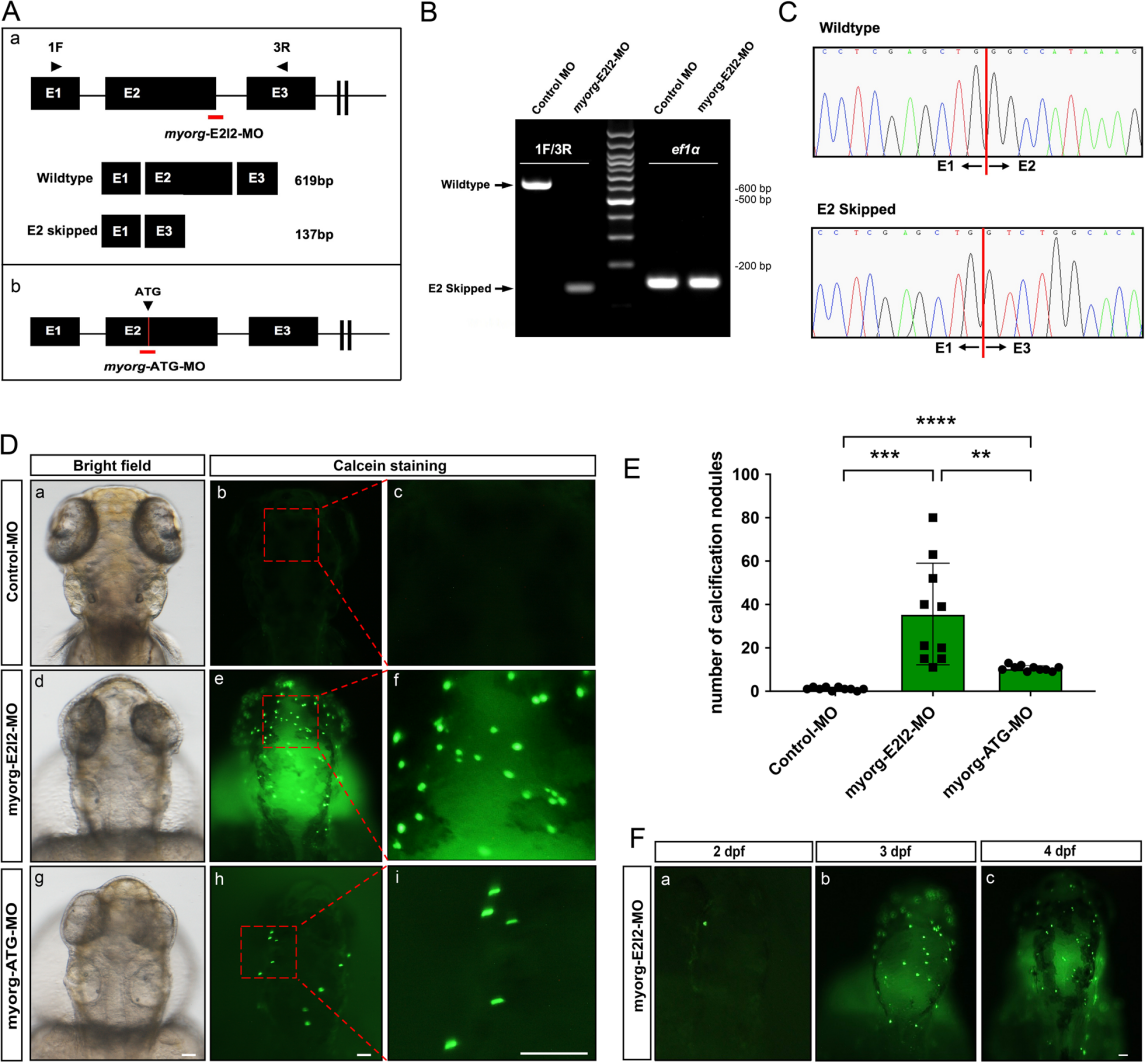
*myorg* knockdown showed brain calcifications in zebrafish. **A** Schematic depiction of the *myorg* transcript after E2I2-MO (**a**) and ATG-MO (**b**) administration. **B** The effectiveness of *myorg* knockdown was confirmed by RT-PCR after morpholino injection of zebrafish larvae at 2 dpf. Injection of 4 ng of *myorg* morpholino altered the splicing between Exon 2 and Intron 2, as revealed by a shift in PCR bands between control (619 bp) and *myorg* morpholino injected embryos (137 bp). **C** Sanger sequencing confirmed the transcription of skipping Exon 2 after the injection of *myorg*-E2I2-MO. dpf, days post-fertilization. **D** Dorsal views of zebrafish embryos injected with control morpholino oligonucleotides (Control-MO), *myorg*-E2I2-MO, and *myorg*-ATG-MO. MO injected embryos were stained with calcein at 3 dpf. Compared to Control-MO (**a–c**), *myorg*-E2I2-MO (**d–f**), and *myorg*-ATG-MO (**g**–**i**) showed potent brain calcifications as green fluorescence in the zebrafish brain. (**a, d, g**) bright field; **(b, e, h)** calcein staining; the magnified zooms of the red dashed box are shown in **c, f, i**. **E** Quantification of the brain calcifications in the *myorg*-E2I2-MO and *myorg*-ATG-MO injected zebrafish at 3 dpf compared to that in Control-MO injected zebrafish. **F** Time-course analysis of brain calcifications in the *myorg*-E2I2-MO knockdown zebrafish. Means ± SEM, n = 10, Student’s *t*-test, ****P < 0.0001, ***P < 0.001, **P < 0.01. Scale bar, 50 μm. *dpf* days post-fertilization

We previously found that *Myorg*-KO mice could develop brain calcifications in the thalamus when they were nine months old. To examine the loss-of-function effect of *myorg* in zebrafish, we designed and injected zebrafish with an antisense oligo targeting Exon 2 and Intron 2 (E2I2-MO) of *myorg* to block its splicing sites (Fig.  2Aa). The effectiveness of *myorg* knockdown was confirmed by PCR of cDNA after morpholino injection of zebrafish larvae at 2 dpf (Fig. 2B). As a comparison, *e1fα* showed similar transcript abundance under both the *myorg-*E2I2-MO and Control-MO intervention (Fig. 2B). Sanger sequencing also validated that E2I2-MO resulted in a complete skipping of Exon 2 in the transcript (Fig. 2C), leading to a knockdown of *myorg* in zebrafish. We also utilized another morpholino targeting the start codon of *myorg* (ATG-MO), in order to prevent the protein expression by blocking its translation initiation (Fig. 2Ab).

We observed the calcifications in the head of *myorg* knockdown zebrafish before skeletal formation around 5 dpf using calcein, a calcium-binding fluorescent chromophore. The fluorescent chromophore, calcein (C_30_H_26_N_2_O_13_), specifically binds to calcium, fluorescently staining the calcified structures and allowing for highly sensitive analysis of brain calcifications in live zebrafish larvae [24]. After microinjection of the *myorg-*E2I2-MO and *myorg*-ATG-MO into fertilized one-cell stage embryos, the calcification number in the *myorg*-knockdown zebrafish brain was 35.6 (11–80) and 10.6 (9–13), respectively, compared to the control group value of 1.1 (0–2) in 3 dpf zebrafish (Fig. 2D, E; Table 1). The number of brain calcification deposits caused by *myorg-*E2I2-MO were more than that by *myorg*-ATG-MO. We also observed that the number of green fluorescent signals was consistently increased during 2–4 dpf after microinjection of the *myorg-*E2I2-MO, while the size of calcification nodules was uniform (Fig. 2F). Indeed, the *myorg*-knockdown zebrafish became weak after *myorg*-E2I2-MO and *myorg*-ATG-MO injection, and they could not survive longer than about one week. Collectively, we confirmed that calcified deposits developed in the *myorg* knockdown zebrafish brains before 5 dpf using two kinds of morpholino-mediated strategies.

**Table 1:** Number of calcification nodules in *myorg* knockdown zebrafish at 3 dpf (10 embryos for each group)

	Control-MO (N = 10)	*myorg*-E2I2-MO (N = 10)	*myorg*-ATG-MO (N = 10)
Number of brain calcification nodules	0	52	10
	2	39	11
	1	63	13
	1	80	11
	2	11	10
	2	20	9
	1	40	12
	1	15	11
	0	15	10
	1	21	9
Mean	1.1	35.6	10.6
SEM	0.2333	7.403	0.4

### *myorg* cDNA rescued the E2I2-MO induced brain calcification

**Fig. 3:**
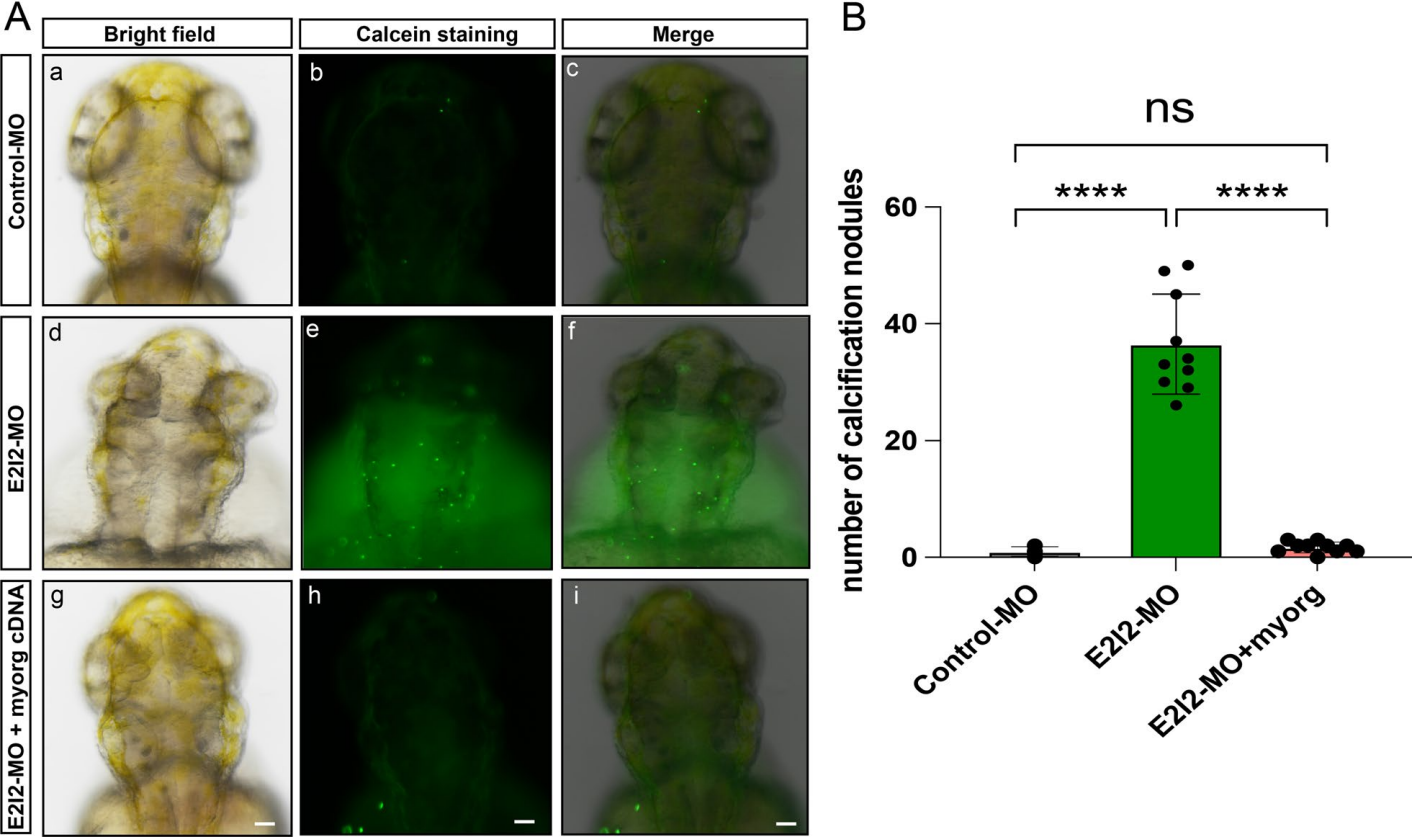
*myorg* cDNA rescued the E2I2-MO induced calcification phenotype. **A** Dorsal views of zebrafish embryos injected with control morpholino oligonucleotides (Control-MO), *myorg*-E2I2-MO, and E2I2-MO combined with *myorg* cDNA. These embryos were stained with calcein at 3 dpf. Compared to Control-MO (**a**–**c**), *myorg*-E2I2-MO showed potent brain calcifications as green fluorescence in the zebrafish brain (**d–f**), E2I2-MO plus *myorg* cDNA resulted in reduced calcification nodules (**g–i**). **a, d, g** bright field; **b, e, h** calcein staining; the merged images are shown in **c, f, i**. **B** Quantification of the calcification number in the brain in *myorg*-E2I2-MO with or without *myorg* cDNA injection to zebrafish at 3 dpf compared to that in Control-MO injected zebrafish. Means ± SEM, n = 10, Student’s *t*-test, ****P < 0.0001. Scale bar, 50 μm

To verify the calcification phenotype using *myorg*-knockdown strategies in zebrafish, we performed the rescue assay to confirm its accuracy. We synthesized the *myorg* cDNA and cloned it into the pcDNA3.1 vector, then co-injected it, along with *myorg*-E2I2-MO, into zebrafish embryos. Compared to the *myorg*-E2I2-MO alone, administering E2I2-MO and *myorg* cDNA plasmid decreased calcification deposits at 3 dpf (Fig. 3A, B). Based on E2I2-MO knockdown, the number of calcification nodules is around 1.7 (0–3) after *myorg* cDNA replenishment, which is close to the effect resulting from Control-MO (Fig. 3B). In conclusion, our data confirmed that knockdown of *myorg* in zebrafish could develop brain calcification, and this phenotype could be mitigated by *myorg* cDNA compensation.

## Discussion

The specific function of MYORG in PFBC remains unclear, meaning that animal models simulating PFBC-like brain calcifications could help elucidate the mechanism of PFBC and relevant therapies. In this study, we demonstrate that knockdown of *myorg* in zebrafish by ASO could successfully mimic the brain calcification phenotype, which could serve as another animal model for assessing *MYORG*-associated brain calcification.

Of the six causative genes, we reported that *MYORG* was the first and most prevalent pathogenic gene in autosomal recessive PFBC [26, 27]. Gene knockout mice were mostly used to mimic the phenotype of PFBC patients. However, neurodegenerative disorders progress relatively slowly, which extends the time required to observe phenotypes such as brain calcifications. Using histological staining, *Slc20a2*-KO mice exhibited relatively obvious calcified nodules at 15 weeks old, which is one of the earliest emerging PFBC mouse models [28]. Similarly, the *Pdgfb*^ret/ret^ mice developed brain calcifications at two months old [9]. We observed the calcified deposits in the thalamus around the nine months old in *Myorg*-KO mice [11]. Nevertheless, no obvious brain calcification phenotypes were present in the *Pdgfrb* and *Jam2* knockout mouse models until they were 14 and 18 months old, respectively [16, 29]. To some extent, our *myorg*-knockdown zebrafish could mimic the brain calcification phenotype at an early age before skull formation, which could accelerate the emergence of the diagnostic phenotype and facilitate mechanistic studies.

Zebrafish can be used to simulate disease-associated phenotypes in mammalian species [30–32]. Using zebrafish as a model has several advantages, including their small size, easy maintenance, fast growth, and short generational time, etc. In particular, embryo zebrafish appear to be transparent, which is advantageous for monitoring the development or pathogenic states of certain organs, such as the complicated central nervous system (CNS), while zebrafish also provide a tractable system for measuring certain behaviors [33]. To date, zebrafish have been used to explore CNS disorders, including autism spectrum disorders, cerebrovascular disorders, neuromuscular diseases, epilepsy, hereditary spastic paraplegia, and neurodegenerative diseases [34–39]. Our findings indicate that brain calcification deposits could be detected in the zebrafish embryos before the skeletal system forms, which could make them a complementary model similar to above-mentioned PFBC mouse models.

Gene knockdown has been widely used to mimic clinical phenotypes in zebrafish [22], especially the antisense oligo blocking strategy. In comparison, gene knockout zebrafish models have been demonstrated to produce genetic compensation response (GCR), which depends on premature termination codons or the homology of transgene sequence with the compensatory endogenous genes [40]. Alleles that are transcribed in response to the deleterious mutation could display more severe phenotypes than alleles with mutant mRNA decay since they can escape the transcriptional adaptation [41]. Morpholino delivery to zebrafish can rapidly model the disease phenotype and potentially avoid the phenotype discrepancies. Knockdown zebrafish models have been used to successfully mimic the typical phenotypes of neutrophil defect syndrome, early-onset stroke and vasculopathy, cerebral small-vessel disease, macular degeneration, adolescent idiopathic scoliosis, and limb-girdle muscular dystrophy [36, 37, 42–45]. We applied the ASO strategy to zebrafish zygotes and observed multiple calcified nodules in the brain parenchyma of *myorg* knockdown zebrafish, and the calcification nodules were persistent at least in 2–4 dpf, successfully modeling the PFBC phenotype. We also rescued the phenotype by supplementing the *myorg* cDNA, which served as a key control to validate the fidelity of brain calcification in *myorg*-knockdown zebrafish. However, the knockdown strategy also has limitations, for example, ASO knockdown effects cannot be steadily passed on to its offspring, and the *myorg*-knockdown zebrafish could not survive more than one week because of the inability to obtain feed. This hinders us from continuously tracking the pathogenic characteristics.

Conventional calcification detection methods have used histochemical stainings including Alcian blue, Alizarin red, and Von Kossa. The widespread calcification in vasculature could be observed in the α-klotho knockout zebrafish at five months old [46]. However, it is also confirmed that Alcian blue and Alizarin red are not sensitive enough to recognize the calcified bone structure in zebrafish embryos [24]. Our *myorg* knockdown larval zebrafish is more fragile with PFA fixing when prepared for immunohistochemical staining, which hinders us from recognizing their physical structures. Accordingly, the results were not stable. Compared to the above two bone markers, calcein staining is more convenient, inclusive, and accurate without toxicity in a live state.

In summary, we established zebrafish as a novel model to simulate brain calcifications in animals other than mice. Our results demonstrate that knockdown of *myorg* by ASO could lead to calcification in the brain of zebrafish embryos. This could promote further study of the molecular mechanisms and precise therapies for PFBC.

## Supplementary information

**Additional file 1: Fig. S1. ***myorg* expression of zebrafish in different developing stages (https://www.ebi.ac.uk/gxa/home).** Table S1.** The mean Ct values for *myorg* and *ef1α* mRNA expression in different regions of adult zebrafish.

## Data Availability

All experimental protocols are described in the “Materials and methods” section or in the references therein, and resources are available upon request from the corresponding authors XPY and WJC.

## Abbreviations

PFBC: Primary familial brain calcification
dpf: Days post-fertilization
ASO: Antisense oligonucleotides
BBB: Blood–brain barrier
CNS: Central nervous system
GCR: Genetic compensation response
