# Supplementary material for: Knockdown of myorg leads to brain calcification in zebrafish
Source: Mol Brain. 2022 Jul 23;15:65. doi: 10.1186/s13041-022-00953-4 (PMC9308368; doi:10.1186/s13041-022-00953-4)
Supplement: Supplementary file 1 — Additional file 1: Fig. S1. myorg expression of zebrafish in different developing stages (https://www.ebi.ac.uk/gxa/home). Table S1. The mean Ct values for myorg and ef1α mRNA expression in different regions of adult zebrafish. [file 13041_2022_953_MOESM1_ESM.docx]

**Supplementary Data**

**Knockdown of *myorg* leads to brain calcification in zebrafish**

**Authors and affiliations:**

Miao Zhao^1,†^, Xiao-Hong Lin^1,†^, Yi-Heng Zeng^1,†^, Hui-Zhen Su^1^, Chong Wang^1^, Kang Yang^1^, Yi-Kun Chen^1^, Bi-Wei Lin^1^, Xiang-Ping Yao^1,*^, Wan-Jin Chen^1,*^

^1^ Department of Neurology and Institute of Neurology of First Affiliated Hospital, Institute of Neuroscience, and Fujian Key Laboratory of Molecular Neurology, Fujian Medical University, Fuzhou 350005, China

^†^ These authors contributed equally to this work.

*Correspondence to: Xiang-Ping Yao, Department of Neurology and Institute of Neurology of First Affiliated Hospital, Institute of Neuroscience, Fujian Key Laboratory of Molecular Neurology, Fujian Medical University, Fuzhou 350005, China.

E-mail address: yaoxiangping@fjmu.edu.cn

*Correspondence to: Wan-Jin Chen, Department of Neurology and Institute of Neurology of First Affiliated Hospital, Institute of Neuroscience, Fujian Key Laboratory of Molecular Neurology, Fujian Medical University, Fuzhou 350005, China.

E-mail address: wanjinchen75@fjmu.edu.cn

**
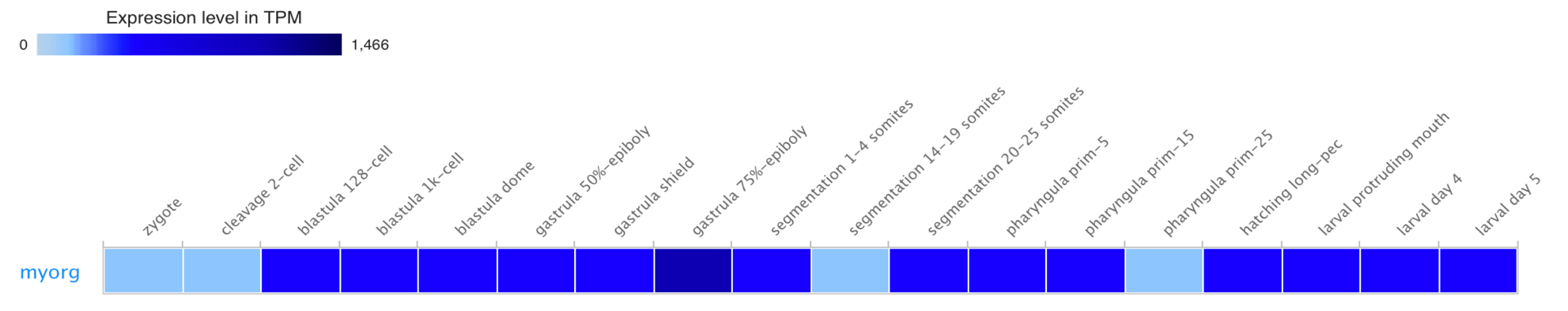
**

**Supplementary Figure 1. *myorg* expression of zebrafish in different developing stages (****https://www.ebi.ac.uk/gxa/home).**

**Supplementary Table 1. The mean Ct values for *myorg* and *ef1α* mRNA expression in different regions of adult zebrafish.**

|  | *myorg* | *ef1α* |
| --- | --- | --- |
| cortex | 28.25 | 18.46 |
| cerebellum | 16.50 | 19.98 |
| hypothalamus | 22.75 | 17.33 |
| medulla oblongata | 28.95 | 23.97 |
| optic tecta | 27.60 | 18.18 |
| liver | 28.73 | 18.26 |
| eyes | 23.74 | 18.13 |
| heart | 29.04 | 16.66 |
| kidney | 26.85 | 20.63 |
| muscle | 23.15 | 25.59 |
| intestine | 21.81 | 15.66 |
